# Fabrication of THz corrugated wakefield structure and its high power test

**DOI:** 10.1038/s41598-023-29997-9

**Published:** 2023-02-24

**Authors:** H. Kong, M. Chung, D. S. Doran, G. Ha, S.-H. Kim, J.-H. Kim, W. Liu, X. Lu, J. Power, J.-M. Seok, S. Shin, J. Shao, C. Whiteford, E. Wisniewski

**Affiliations:** 1 Pohang Accelerator Laboratory, POSTECH, Pohang, Gyungbuk 37673 Korea; 2 Department of Physics, Kyungpook National University, Daegu, 41566 Korea; 3 Ulsan National Institute of Science and Technology, Ulsan, 44919 Korea; 4 Argonne National Laboratory, Argonne, IL 60439 USA; 5 Northern Illinois University, Dekalb, IL 60115 USA; 6 Department of Accelerator Science, Korea University, Sejong, 30019 Korea

**Keywords:** Mechanical engineering, Applied physics, Techniques and instrumentation

## Abstract

We present overall process for developing terahertz (THz) corrugated structure and its beam-based measurement results. 0.2-THz corrugated structures were fabricated by die stamping method as the first step demonstration towards GW THz radiation source and GV/m THz wakefield accelerator. 150-$\upmu$m thick disks were produced from an OFHC (C10100) foil by stamping. Two types of disks were stacked alternately to form 46 mm structure with $\sim$ 170 corrugations. Custom assembly was designed to provide diffusion bonding with a high precision alignment of disks. The compliance of the fabricated structure have been verified through beam-based wakefield measurement at Argonne Wakefield Accelerator Facility. Both measured longitudinal and transverse wakefield showed good agreement with simulated wakefields. Measured peak gradients, 9.4 MV/m/nC for a long single bunch and 35.4 MV/m/nC for a four bunch trains, showed good agreement with the simulation.

## Introduction

In order to overcome the primary limit of conventional linear accelerator, the Advanced Accelerator Concepts (AAC) have been proposed and demonstrated for realizing future energy-frontier colliders^1–6^ and compact multi-beamlines X-ray free electron lasers^7–9^. Structure wakefield acceleration (SWFA) is one of the AAC that utilizes particle beams (= I) and high impedance structures (= R) to generate intense electromagnetic fields (= V) called wakefield^10,11^. This intense wakefield can either accelerate particle beams with a high accelerating gradient or irradiate targets for various purposes (e.g., particle acceleration^12^, THz pump-probe^13^, non-destructive examination^14^, etc.).

Recently significant progress has been made by Pohang Accelerator Laboratory and Argonne National Laboratory. While most SWFA researches have been done in tens of gigahertz regime [AWA], we demonstrated the fabrication and high power test of a terahertz (THz) structure which has begun to attract increasing attention due to its feasibility of reaching gigawatt or GV/m class^12,15–17^. We fabricated a cylindrical corrugated structure, which is one of the most representative structure for SWFA. As the first step towards gigawatt and GV/m (i.e., THz-SWFA with relaxed dimensions), $\sim$ 0.2 THz corrugated structure was fabricated by die stamping method.

**Figure 1:**
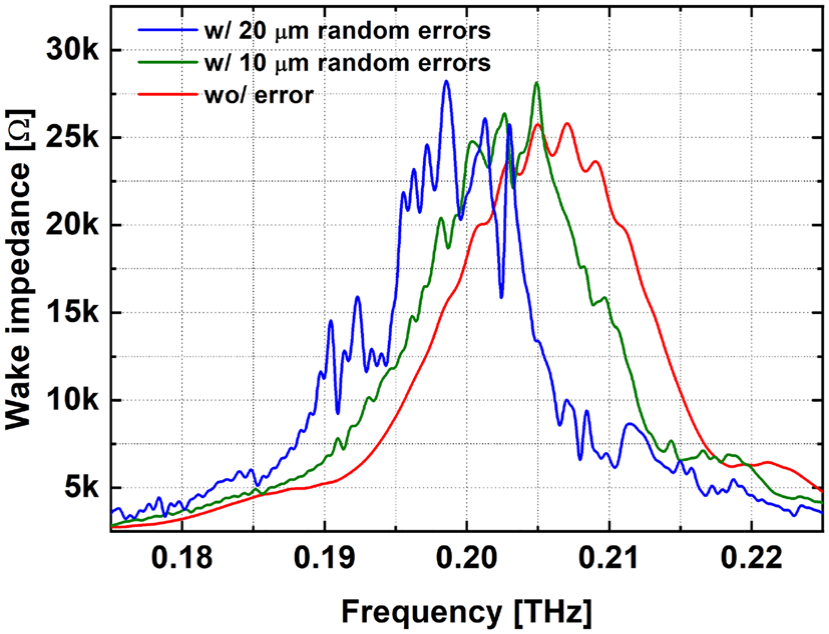
Tolerance simulation by CST particle studio. Machining errors and transverse offsets of each disk are randomly provided within the given range. The reference case (red) shows symmetrical peaks around 0.206 THz. The random errors with 10 $\upmu$m (green) and 20 $\upmu$m (blue) show shifted and unpredictable spectrums.

Die stamping method creates two rings forming a single period of the corrugation by stamping of a copper foil. It is more appropriate new method for fabricating THz corrugated structures than conventional methods due to the structure’s remarkably large number of tiny corrugations. While a conventional accelerating column has at most 20 irises (e.g., 1-m long L-band accelerating column has 7 irises^18^), the structure we fabricated has $\sim$ 170 corrugations in 46 mm. Here, the quality of the corrugation (e.g. machining error, perpendicularity, concentricity) has significant impact on the wakefield inside the structure; see Fig. 1. The structures with errors shown in Fig. 1 are examples. However, they show that the errors should be as small as possible and the tolerance is less than 0.5$\%$ of the aperture size. It is difficult to produce such a large number of tiny corrugations with high-precision using conventional methods such as shrinking, electroforming, and brazing^19,20^. On the other hand, the die stamping method easily produces a large number of disks and controls the quality of each disk.

## Results

### Structure design

**Figure 2:**
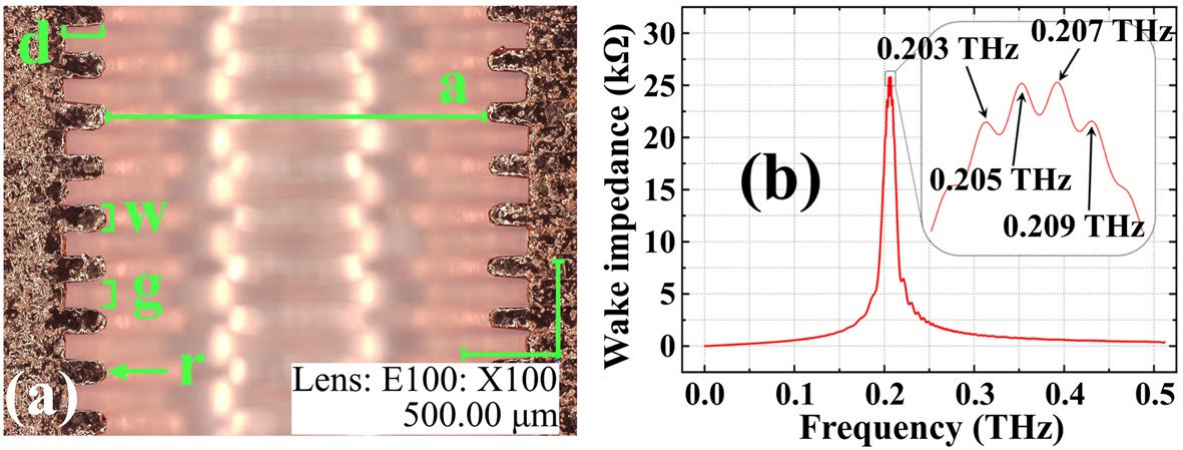
Fabricated corrugated structure and corresponding simulated wake impedance. (**a**) A picture of a half-cut accelerator column. Note that the structure in the picture is not the high-power-tested one. Although the design was the same, the final dimension could have difference due to the polishing process. (**b**) Longitudinal wake impedance of the optimized design. Impedance peaks are located in 0.20–0.21 THz.

The structure was designed to achieve a single-mode and a high-gradient wakefields. We optimized structure parameters displayed in Fig. 2a; corrugation depth (d), corrugation width (w), gap between corrugations (g) and the rounding radius (r). The aperture (a) was fixed to 2 mm to ease the particle-beam-transport for the high-power test. CST microwave studio^21^ and particle studio^22^ were used for the optimization. During the optimization, we used a ultra-relativistic Gaussian bunch whose root-mean-square (rms) bunch length is 0.2 mm. Table 1 and Fig. 2b show the optimized dimension and corresponding wake impedance, respectively.

**Table 1:** Optimized structure dimensions and structure dimensions for ECHO2D simulation.

Parameter	Optimized dimension (mm)	ECHO2D dimensions (mm)
a	2.00	2.04
d	0.20	0.20
g	0.15	0.13
w	0.15	0.13
r	0.05	0.065

### Structure fabrication

To fabricate the structure, we produced two types of disks that have the same outer diameters (OD) but different inner diameters (ID) by die stamping. An OFHC (C10100) sheet was used to manufacture the disks, and the total number of disks for 1 structure were 355. These disks were stacked alternately to form the corrugation in Fig. 2. As mentioned earlier, the fabrication quality has significant impact on the structure’s performance. Disk alignment error is one of the quality factors. To ensure the perfect contact and alignment of the disks, disks were stacked inside a commercial SS304 pipe. The copper disks’ OD were manufactured to be slightly larger than the ID of the pipe. Then, disks were chemically polished to fit in the pipe. The OD changed by the polishing depends on the polishing time, the temperature and concentration of the acid mixture. Thus, the polishing time was controlled to make the gap between the disk and the pipe within 10 $\upmu$m. The chemical polishing changed other dimensions of disks approximately 20–30 $\upmu$m. Such change would introduce the frequency shift of a few GHz.

It is worth to note that several new methods for fabricating the THz corrugated structure have been proposed and surveyed recently^23^. The die stamping method was one of them. The survey pointed out that the precision cutting along the inner circumference and alignment of large number of disks, which are necessary for the die stamping, are challenging. Thus, we pressed the inner and outer edges multiple times for stamping instead of single-press stamping. Also, edges were pressed in both upward and downward so that the stamped edges can be symmetric. In addition, disks were chemically polished to clear edges from the stamping and to make better alignment and contact between disks.

**Figure 3:**
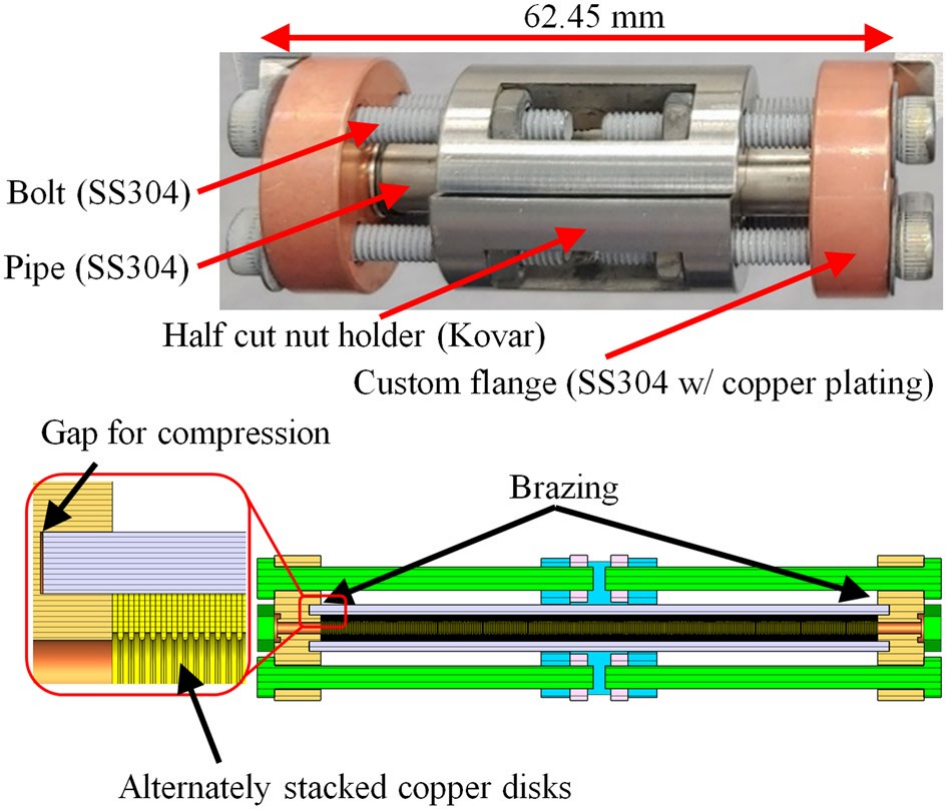
Picture and schematic of a structure assembly for diffusion bonding.

Disks are not flat but slightly concave because they were made by die stamping. To make the tight and flat contacts between the disks without voids, it is necessary to press with an appropriate force. We designed an assembly to press the disks as shown in Fig. 3. Two half cut holders made by kovar^24^ are assembled with two custom flanges at the both end using 8 SS304 bolts. The thermal expansion of the kovar is smaller than both copper and SS304. Thus, the thermal expansion of the 8 bolts and Kovar holders is smaller than the expansion of the stacked disks and flanges. As the bolts hold flanges, flanges press the disks, which guarantees tight and flat contacts without voids. Note that the length of the structure was 62.45 mm. When the temperature rose to 820 $^\circ$C, the thermal expansion of copper disks+flanges was 0.93 mm. On the other hand, the thermal expansion of bolts+holders was 0.77 mm. The high temperature and the pressure diffusion-bonded copper disks and brazed the custom SS304 flanges to the pipe. This brazed flanges ensure the structure assembly’s vacuum-tight condition.

### Setup for electron-beam-based high-power test

The fabricated structure was experimentally examined by using electron beams. We measured longitudinal and transverse wakefields and compared them to simulations. The longitudinal wakefield was measured by the projection technique^25,26^ that allows time-resolved measurement of the longitudinal wakefield. For the transverse wakefield measurement, we introduced a new projection technique that is similar to the longitudinal measurement so that the time-resolved information can be obtained.

**Figure 4:**
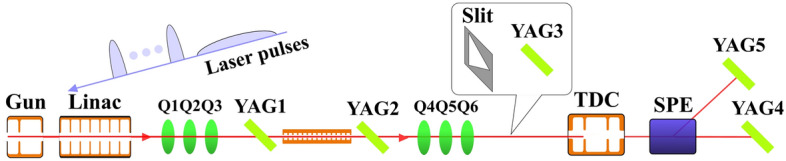
Schematic diagram of experimental beamline.

One of the keys for the projection technique is simultaneously introducing a beam driving wakefields and a long probe beam. In this way, the strength of the wakefield can be evaluated from the beam’s energy or transverse position changes. Ultraviolet laser was split to two laser pulses using the beam splitter to generate the drive and probe electron beams. The source laser pulse’s temporal length was about 300 fs root-mean-square (rms). Because no other electron beam compression mechanism existed in the beamline (see Fig. 4, the 300 fs pulse was directly used for generating the drive beam while five $\alpha$-BBO crystals were introduced to elongate a laser pulse length for the probe beam to 6 ps^27^. The laser path for the probe beam also included a motorized delay line to control its relative timing to the drive laser pulse. Total three cycles of wakefields behind the drive beam were measured using this delay control feature.

The drive and probe beams were accelerated by L-band rf accelerating cavities. Beams’ energies were increased to 45.2 MeV. Three quadrupole magnets were located in front of the corrugated structure to focus beams in both x- and y-planes. Three Yttrium Aluminum Garnet (YAG) screens were installed along the beam path (see Fig. 4) to evaluate the beam envelope. The downstream of the structure was diagnostic area for the wakefield measurement. Structure was attached to a motorized actuator so that it can be inserted and extracted during the experiment. The support system had a rail to secure structure’s vertical position during the insertion and extraction.

A transverse deflecting cavity (TDC) and a rectangular dipole magnet (SPE) were employed to project beam’s temporal and spectral distributions to a YAG screen (YAG5). Quadrupole magnets in front of TDC transversely focus the beam to maximize the resolution. A horizontal slit was located in front of TDC to minimize energy spread growth by Panofsky–Wenzel effect^26^.

### High-power test via wakefield measurement: longitudinal wakefield

**Figure 5:**
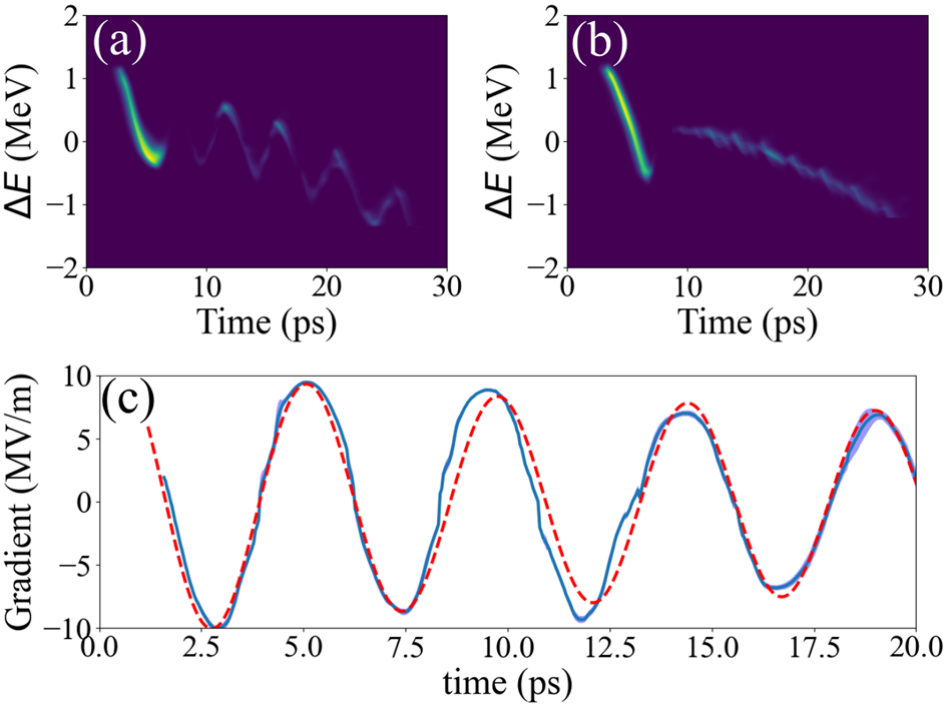
Time-resolved longitudianl wakefield measurement. (**a**,**b**) Longitudinal phase spaces with and without the corrugated structure, respectively. (**c**) The comparison of the measured longitudinal wakefield (blue-solid) and the simulated wakefield (red-dash). The blue shade corresponds to $\pm 1\sigma$ statistical error.

Figure 5’s panel a and b show measured longitudinal phase spaces with and without the structure, respectively. Here we combined snapshots from each probe-beam delay position to provide entire 3-cycle view. The actual energy change in the probe beam can be obtained by subtracting time-energy correlation curves with and without the structure. Because the beam is ultra-relativistic ($\beta =0.99994$), it is fair to assume that the beam’s longitudinal current profile does not change within the structure length of 46 mm. Thus, the energy change divided by the structure length can be considered as the wakefield’s accelerating gradient. The result is displayed in Fig. 5c (blue-solid). Drive beam’s charge was $0.992 \pm 0.004$ nC, and its rms bunch length and corresponding bunch form factor, which is Fourier transform of the current profile, were 1.1 ps rms and 0.3 respectively. Note that the accelerating gradient is proportional to the charge and the form factor.

The maximum energy loss in the drive beam was 0.14 MeV, and the maximum energy gain behind the drive beam was 0.43 MeV. Here the probe beam’s charge is low enough to ignore the beam-loading effect. The structure length, drive beam’s charge, and maximum energy gain provide the peak accelerating gradient of the wakefield. It was 9.4 MV/m/nC.

The wakefield from any charge distribution can be expressed as convolution of the charge distribution and the wake function that is a wakefield from a single electron^28^. Thus, we calculated the wakefield using the measured drive beam’s current profile and a wake function from a simulation code called ECHO2D^29^. Because the measurement was accompanied with a large radial offset, the beam excited higher order azimuthal modes (HOMs). We assumed an constant offset of 480 $\upmu$m in the simulation. The simulation result is shown as red-dash curve in the panel c, and it shows good agreement with the measurement.

Expected 200–210 GHz impedance peaks were shifted, and the 216 GHz frequency dominantly determined the wakefield’s shape. Due to the polishing process, the optimized dimensions in Table 1 could be changed as mentioned earlier. Thus, we have explored parameter space and obtained a reasonable dimension that is within the expected dimension error range and provides good agreement with the measured wakefield. Dimension used for the simulation is given in the third column of Table 1.

### High-power test via wakefield measurement: transverse wakefield

Transverse wakefield is another important factor characterizing the beam transport in the structure. Quantitative measurement of transverse wakefield is not straightforward because both the drive and probe beams travel on the same axis. Conventional technique introduces a long enough space after the structure so that the momentum change from the wakefield generates the probe beam’s transverse offset. However, this method does not work if the kick is weak or travel distance is not long enough. Thus, we tried a new technique to obtain a snapshot of beam’s t–x distribution (x is the plane that we apply the offset so that the transverse wakefield kick the beam).

**Figure 6:**
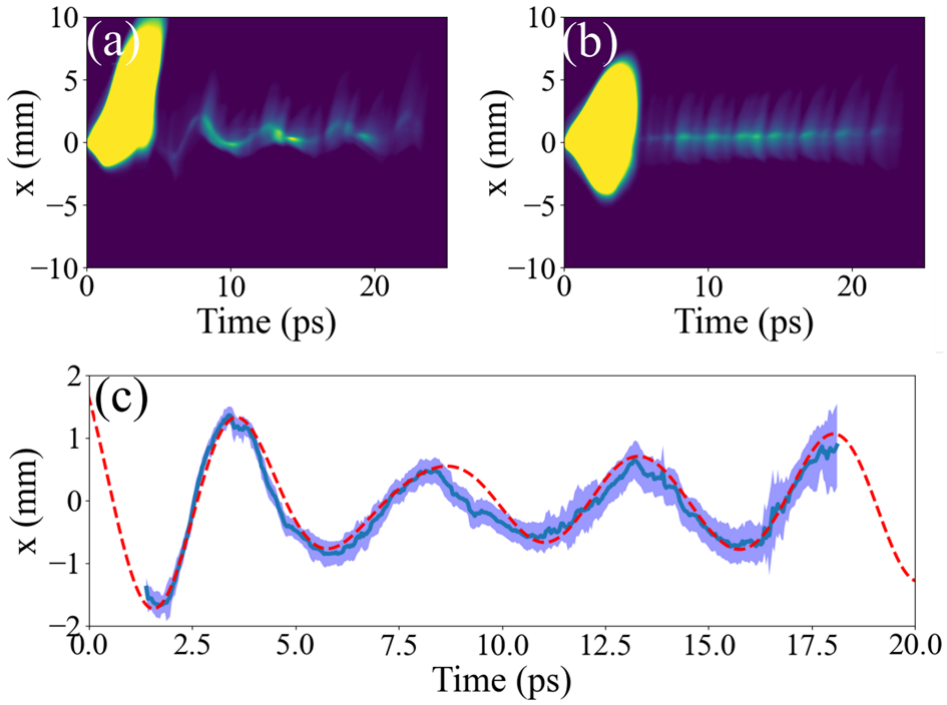
Time-resolved transverse wakefield measurement. (**a**,**b**) Beam’s x–t distribution with and without the corrugated structure, respectively. (**c**) The comparison of the measured transverse wakefield (blue-solid) and the simulated wakefield (red-dash). The blue shade corresponds to $\pm 1\sigma$ statistical error.

We projected beam’s temporal distribution to y-plane using TDC. Contrary to the longitudinal measurement, the dipole magnet was turned off and quadrupole magnets (Q4–6) projected beam’s transverse momentum at the exit of the structure to the YAG screen (YAG4). Measured beam’s t–x distribution with and without the structure are displayed in Fig. 6 a and b, respectively. The panel a is the case that the beam path had an horizontal offset of 480 $\upmu$m from the reference horizontal position where minimizes the transverse wakefield. Similar to the longitudinal measurement, we can achieve the transverse wakefield by subtracting t–x correlation curve from two images.

Note that the particle transport can be expressed as,

1 $$\begin{aligned} x_f = R_{11} x_i + R_{12} x'_i, \end{aligned}$$

where *x* is particle’s horizontal position and $x'$ is its divergence. The quadrupole setting was incomplete to zero $R_{11}$ term, which is the imaging condition for the transverse wakefield. The measured transfer matrix parameters were R11 and R12 was 4.7 and 0.52, respectively. Thus, remained x-term’s effect is combined in the image. We simulated the beam transport with expected horizontal and vertical offsets and applied the measured transfer matrix to estimate projected image at the YAG screen to avoid the confusion. The result is shown in the panel c. The blue curve is the measured transverse wakefield and the red-dash curve is the one from the simulation. Measured and simulated wakefields show good agreement. Similar to the longitudinal measurement, we had a large offset of 480 $\upmu$m. This is large enough to introduce strong HOMs. Here the ratio of HOMs are expected to 1.00:0.56:0.24 (octupole or higher modes were ignorable). These strong HOMs introduced a beating that we can observed from both the measurement and simulation; see Fig. 6.

### High-power test via wakefield measurement: superposition of longitudinal wakefield

**Figure 7:**
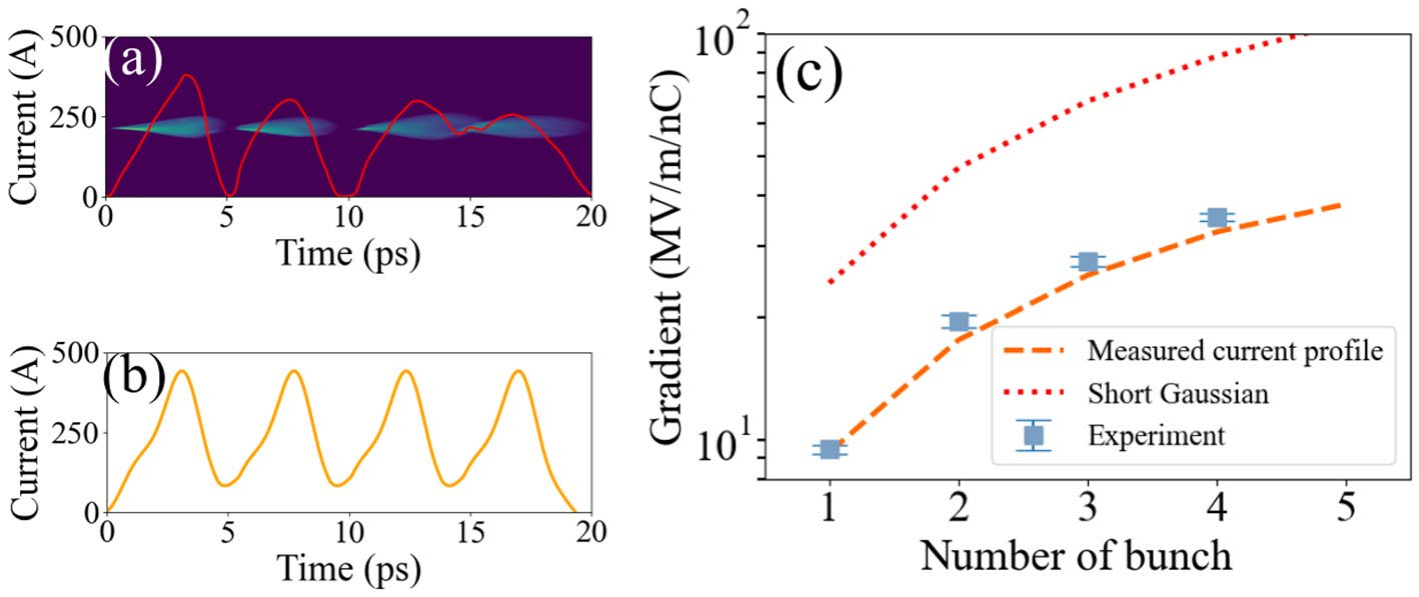
Scaling of achievable gradient from the fabricated structure. Gradients for bunch train were measured up to four bunches (blue-dot, panel **c**), and the corresponding measured current profile is in panel (**a**). “Measured profile” case (red-dash) in panel (**c**) shows the simulated achievable gradient. For this simulation, the measured current profile for a single bunch was duplicated as given in panel (**b**). “Short Gaussian” case (red-dot) in panel c shows the simulation with a short Gaussian that provides the bunch form factor of  1.

The superposition of the wakefield was also examined. Due to a high group velocity ($\approx$ 0.63c), the wakefield damps quickly. Thus, the superposed wakefield’s gradient is not proportional to the number of bunches. We introduced up to four bunches to confirm this nonlinear response. The measured current profile and gradients are shown in Fig. 7 panel a and c, respectively. Note that the charge ratio between bunches was 1:0.77:1.07:1. This is the reason why the third and fourth bunches overlapped.

This current profile measurement for four bunches could have O(0.1)-ps level error because each bunch has different transverse optics which significantly limits the measurement’s resolution^25^. Thus, simulation was performed with a measured current profile from a single drive beam for comparison. For two or more bunches, we duplicated the single beam current profile with appropriate separation that maximizes the gradient. Panel b shows this duplicated current profile for the four-bunch case. Here the separation between bunches was set to 4.62 ps. “measured current profile” (orange-dash) corresponds to this simulation case, and it shows good agreement with the measurement data.

## Discussion

We fabricated a THz corrugated structure using the die stamping method and measured wakefields from the structure. Two types of disks were mass-produced and diffusion bonded to form tiny corrugations. We adopted existing projection technique for the longitudinal measurement and introduced a new projection technique for the transverse measurement. The comparison proved that method’s two challenging points, precise stamping and alignment, were successfully overcame by chemical polishing, external pipe for guiding, diffusion bonding with a pressing assembly.

The structure provided the maximum accelerating gradient of 9.4 MV/m/nC for a single bunch and 35.4 MV/m/nC for four bunches. This gradient can be further increased if short bunches that has a bunch form factor close to 1.0. The expectation for the high form factor case is given in Fig. 7. It is expected to achieve over 100 MV/m/nC with 5 bunches when the form factor is maximized. The fabrication technique we demonstrated is not limited to sub-THz structures or corrugated structures. The experiment result is significant step towards 1.2 GW or 4.2 GV/m wakefield generation from a 1.4 THz structure, which is from previous study^30^.

The die stamping method can also be applied to any other structures having complex geometries like deep-corrugation^31^, metamaterial^32^, HOM suppression geometry^33^. Further development of the technique for dielectric material could also open interesting path towards dielectric-disk structure^34^, dielectric assisted structure^35^, and correlation control via multi-frequency structures^36,37^, which would be promising candidates for high-power THz power sources that enables future TeV linear colliders for high energy physics research, GeV-class accelerator-based light sources supporting various basic sciences.

## Methods

### Wake function from ECHO2D

While wakefield from a single electron is required to calculate wakefield from a beam, it is impossible to simulate a single electron accurately using conventional simulation codes. However, the distribution’s impact becomes negligible when the bunch length is around 1% of the wakefield’s wavelength. Thus, we generated a short bunch whose bunch length is comparable to the simulation step size to minimize the impact from the distribution. The wake potential from this short bunch was normalized by the charge and the structure’s length so that it can be considered as the wake function (i.e., wakefield from a single electron).

### Wakefield simulation for comparison

The ECHO2D simulation returns longitudinal wakefields for defined azimuthal modes. We included the first four azimuthal modes because the magnitude of the fifth mode is less than 1% of the first mode that is dominant. Transverse wakefields were calculated from the longitudinal wakefields using the Panofsky–Wenzel theorem. For the comparison, we generated and tracked a pencil beam. The transverse distribution of the beam was ignored due to transverse wakefield’s negligible impact on the particle position shifts (less than 50 $\upmu$m). On the other hand, a current profile of the beam determines wakefield’s major characteristics. Thus, we imported measured current profiles as the simulation input. Simulation step size was 100 $\upmu$m. At each time step, we convoluted the present current profile with the wake function.

## Data Availability

The data that supports the result of this demonstration are available from the corresponding authors upon reasonable request.
