# Author Correction: Fabrication of THz corrugated wakefield structure and its high power test

**DOI:** 10.1038/s41598-023-31386-1

**Published:** 2023-03-14

**Authors:** H. Kong, M. Chung, D. S. Doran, G. Ha, S.-H. Kim, J.-H. Kim, W. Liu, X. Lu, J. Power, J.-M. Seok, S. Shin, J. Shao, C. Whiteford, E. Wisniewski

**Affiliations:** 1 Pohang Accelerator Laboratory, POSTECH, Pohang, Gyungbuk 37673 Korea; 2 Department of Physics, Kyungpook National University, Daegu, 41566 Korea; 3 Ulsan National Institute of Science and Technology, Ulsan, 44919 Korea; 4 Argonne National Laboratory, Argonne, IL 60439 USA; 5 Northern Illinois University, Dekalb, IL 60115 USA; 6 Department of Accelerator Science, Korea University, Sejong, 30019 Korea

Correction to: *Scientific Reports* 10.1038/s41598-023-29997-9, published online 24 February 2023

In the original version of this Article, author G. Ha was omitted as a corresponding author. Correspondence and requests for materials should also be addressed to gwanghui.ha@gmail.com.

The original Article has been corrected.

